# Histopathology of Cervical Cancer and Arsenic Concentration in Well Water: An Ecological Analysis

**DOI:** 10.3390/ijerph14101185

**Published:** 2017-10-06

**Authors:** Mohammad Golam Mostafa, Zarat Jahin Queen, Nicola Cherry

**Affiliations:** 1 National Institute of Cancer Research and Hospital, Dhaka 1206, Bangladesh; dr.mohammadgolammostafa@gmail.com; 2 Manchester Medical School, University of Manchester, Manchester M13 9PL, UK; queenzj.2502@gmail.com; 3 Department of Medicine, University of Alberta, Edmonton, AB T6G 2R3, Canada

**Keywords:** Bangladesh, cervical cancer, arsenic, poorly differentiated squamous cell, epidemiology

## Abstract

Arsenic in drinking water is causally linked with cancer of the skin, lung, and urinary bladder, but there is very little data on a possible role for arsenic in the etiology of cervical cancer, a disease in which human papilloma virus is held to be a necessary but not sufficient cause. All histopathology results from cervical specimens from the National Institute of Cancer Research and Hospital (NICRH), Dhaka (1997–2015), and the Anowara Medical Services (2003–2015), both serving the whole of Bangladesh, were classified by cell type. Arsenic concentrations in well water in the thana of residence were estimated from systematic sampling carried out by the British Geological Survey. In a case-referent analysis arsenic estimates for cases of cervical cancer were compared with those found to have benign lesions. In this study, 3464 NICRH (CH) cervical specimens and 30,050 community medical service (CMS) specimens were available: 3329 (CH) and 899 (CMS) were recorded as malignant. Most were squamous cell carcinoma, of which 4.9% were poorly differentiated. Overall, there was no increase in cervical cancer with increasing arsenic concentration. Among those with squamous cell histology, a strong dose response was seen for poorly differentiated cancer with increasing arsenic exposure. The odds ratio increased monotonically, compared with exposure <10 μg/L, from 1.58 at 10 < 50 μg/L to 8.11 at >200 μg/L (*p* < 0.001). Given the high proportion of Bangladeshis using drinking water containing >50 μg/L of arsenic, the evidence that arsenic is implicated in cancer grade suggests a need for further investigation and the introduction of cervical screening in high arsenic areas.

## 1. Introduction

Although it is well established that arsenic in drinking water is a causal factor in cancers of the urinary bladder, lung, and skin [1], and that data for renal cancer is suggestive of causality [2], there has been almost no investigation of a possible link between arsenic exposure and cancer of the uterine cervix (cervical cancer), the second most frequent female cancer in most Asian countries [3,4]. Wu et al. [5] included six deaths from cervical cancer in their study of arsenic related mortality in 42 villages in Taiwan. Although the number was too small to reach any conclusion, they reported an increase in standardized mortality ratio with higher arsenic exposures. One study from India investigated the relation between arsenic exposure and oxidative stress (as reflected in malondialderhyde (MDA)) in cervical cancer cases and referents and reported higher levels of arsenic and MDA in cases [6]. In Bangladesh, a high proportion of the population is exposed to arsenic in drinking water, with 27% of hand-pumped tube wells tested by the British Geological Survey, commissioned by the People’s Republic of Bangladesh, found to have arsenic concentration >50 μg/L [7]. These wells clustered particularly in areas south and east of the capital (Dhaka), but with arsenic “hot spots” throughout the country.

Since it has been concluded that human papilloma virus (HPV) is a necessary but not sufficient cause for cervical cancer [8] and that population screening and HPV vaccination are effective means of control, there has been limited investigation of risk factors that may influence the progress to clinical disease: multiple pregnancies, oral contraception, tobacco use, and immunocompromise (particularly from human immunodeficiency virus, HIV) are known to increase the risk of invasive cervical cancer [8]. Other factors such as multiple sexual partners (in the woman or her partner) increase risk of cervical cancer by increasing the likelihood of HPV infection. In Bangladesh, there is only very limited information on HPV infection [9,10,11,12], and, together with other Asian countries, there is a relatively low cumulative incidence of cervical cancer (close to 2%) [4,12]. There is currently little cervical screening or HPV vaccination among Bangladeshi women [4,12]. The pattern of risk factors that may alter the incidence or the course of the disease differs from those in more developed countries: in Bangladesh, the age of marriage has traditionally been low, and the number of pregnancies high. In recent years, oral contraception has been widely used. HIV rates are low [12]. There is little use of smoked tobacco among women, but around one in four may use smokeless tobacco in betel quid or paan [13].

Although there is consensus that arsenic in drinking water is a cause of some cancers there is less certainty about the mechanism. In reviewing experimental studies (on rodents) IARC concluded that “in multiple studies, initiating, promoting or co-carcinogenic activity was demonstrated in the urinary bladder, skin, female reproductive tract, kidney, lung, liver and thyroid after exposure to inorganic arsinicals or DMA in drinking water” [2]. In the human epidemiology of arsenic and cancer, the pattern of metabolism and excretion appears important, with a higher proportion of methylated arsenic in urine being a marker of higher risk of skin or urinary cancer [14]. It has been suggested that arsenic acts through an epigenetic mechanism, with exposure leading to DNA methylation regulating gene expression [15], and as a source of oxidative stress [6]. Given that arsenic is implicated in cancer causality and that other extrinsic toxins such as tobacco appear to increase the risk of invasive cervical cancer, the opportunity was taken, using methods previously developed to investigate lung, renal and bladder cancer [16,17,18], to examine whether residence in an area with high arsenic concentration in well water was associated with a higher risk of cervical cancer.

## 2. Methods

Information was available from 2 sources. The National Institute of Cancer Research and Hospital (NICRH) (CH) series included data for all cases from 1997 to 2015 in which cervical specimens had been examined for patients seen at or admitted to the NICRH in Dhaka, Bangladesh. The Community Medical Service (CMS) series comprised histopathology and cytology results from 2003 to 2015 recorded for cervical specimens analysed at the Anowara Medical Service, in Dhaka, which provides a service of histopathology and cytology, poorly accessible elsewhere in Bangladesh. In 2011, about 20% of all tissue diagnoses in Bangladesh were made in this clinic at which specimens are assessed from patients all over the country. The specimens submitted were collected because of some level of clinical suspicion, including cases in which a biopsy was taken following a positive visual inspection with acetic acid (VIA). The diagnoses in both series were carried out in all time periods under the direction of the first author (MGM). The analysis was limited to women age 18 years and older at the time the sample was taken.

Duplicates with the same case number were removed from the CMS records: if more than one sample was present for the same woman and the diagnoses differed, one being malignant, the malignant sample only was retained.

Arsenic exposure was estimated from concentrations in drinking water in the thana (the smallest administrative area within Bangladesh) in which the woman was living at diagnosis. Systematic sampling of 3534 wells by the British Geological Survey in 1998–1999 provided estimates by thana for 61 of the 64 districts into which Bangladesh was divided at that time. No estimates were made for three districts in the remote Chittagong Hill Tracts. Arsenic concentrations were considered initially as <10 μg/L, ≥10 <50 μg/L), ≥50 <100 μg/L, and ≥100 μg/L. Non-detected values were replaced by 0.05 μg/L.

In this analysis there were many unmeasured confounders, with only age, year of diagnosis and place of diagnosis (CH or CMS) extracted from the record. For this study of cervical cancer, in contrast to previous studies of lung [16], renal [17], and bladder [18] cancer using these exposure estimates, individual data on use of well water, length of residence in a village, and tobacco habit were not available.

## 3. Statistical Methods

A case-referent (case-control) approach was used. Initially exposure (the estimated arsenic concentration in well water) was compared for cases (each pre-malignant or malignant condition in turn) with that of an internal referent group, those with benign diagnoses from the same source (CH or CMS). Chi-square tests were used to test for differences in distributions and to test for linearity in the cross tabulations.

As there was some possibility of selection bias in the internal referent groups, an additional referent series, specimens from women with benign renal lesions submitted to the same community medical service in 2008–2011, was used to compare arsenic distributions. These women had biopsies because of suspicion of renal cancer but with lesions that proved to be benign [17]. A multilevel logistic regression, allowing for clustering within thana, was used to investigate the relationship of all malignancies and squamous cell cancers to arsenic concentration using the external referent group. An internal, subgroup analysis examined factors associated with a histological diagnosis of poorly differentiated squamous cell carcinoma among those with a squamous cell type.

## 4. Results

**Figure 1:**
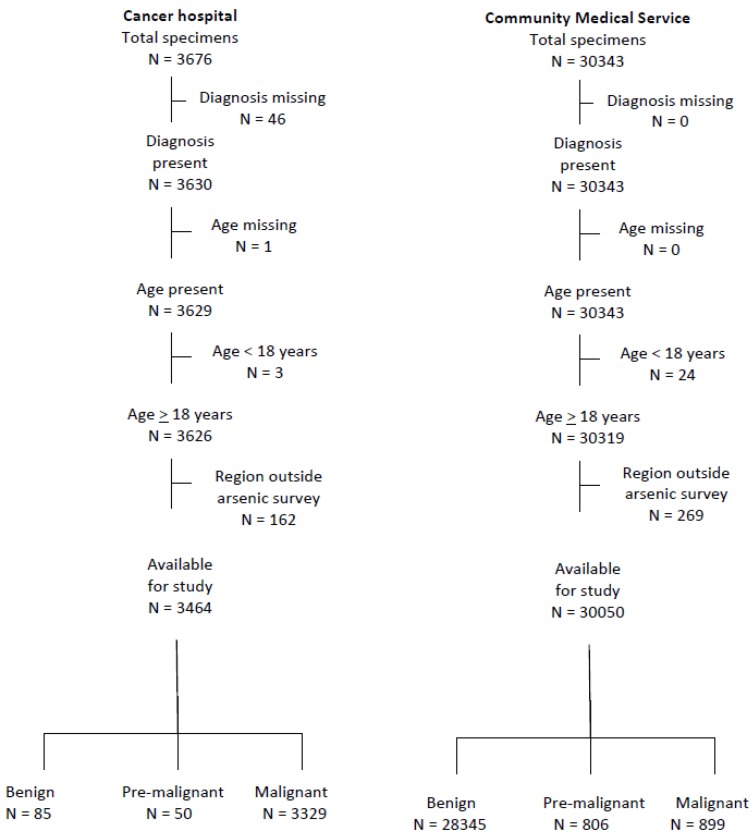
Study population.

As can be seen from Figure 1, 3464 specimens from cancer hospital patients and 30,050 of those from the community medical service were available for analysis. Among those from the cancer hospital, only 85 (2.5%) were recorded as benign, but 94.3% (28,345/30,050) of those from the CMS showed no malignant or pre-malignant condition. The cell type reported for the malignancies was most frequently squamous cell in both series (Table 1), of which 95% were well differentiated. Adenocarcinomas and other malignancies were recorded more frequently in the community series, together accounting for close to 17% of malignancies. In both series, the mean age of those with benign and premalignant lesions was lower than those with malignant lesions. In the cancer hospital series, those with adenocarcinoma tended to be younger and those with benign lesions to have more recent specimens. In both series, there was little difference in age between those with poorly differentiated and well differentiated squamous cell lesions (overall, *p* = 0.638), but those with poorly differentiated cancers tended to have an earlier year of diagnosis.

Patients from the cancer hospital with a diagnosis of cervical malignancy (N = 3329) were drawn from all 61 districts, in which the British Geological Survey had determined arsenic levels, and from 430 thanas. The smaller number from the CMS (N = 899) were drawn from 58 of the 61 districts (208 thanas), supporting the expectation that both series would include women from throughout Bangladesh. The relation between cell type and arsenic concentration in wells within the thana of residence is shown in Table 2. The distribution by arsenic concentration for each malignant and pre-malignant diagnosis was tested against the distribution of arsenic, within each series, for those found to have a benign lesion within the same series. If there were a dose-response relationship between arsenic concentration and the malignancy, increasing malignancy with concentration would be reflected in a linear trend. Such a trend is seen, against the internal referent group, only for squamous cell carcinomas in the cancer hospital series.

There was no certainty that either internal referent series was unbiased with respect to the geographic area from which they were drawn. At the NICRH, the number was small, and the pathology specimens recent, reflecting a change in admissions policy. In the community series, a large but unknown proportion was drawn from symptom based screening clinics at medical colleges or certain district level hospitals that do not cover the whole population of Bangladeshi women. To further evaluate the largely negative relation between malignancy and arsenic concentration, an alternative, external comparison series was also considered, using data from the same CMS [17]. The 222 women with benign renal lesions were from all 61 districts covered by the BGS estimates of arsenic exposure. The arsenic concentrations for this external referent series are also included in Table 2.

Overall, those with any cervical malignancy were less likely than those in the external referent group to live in a thana with estimated arsenic concentration ≥100 μg/L (Table 3). Within cell type, the only differences seen were the decreased rate of well differentiated squamous cell lesions and the increased number of poorly differentiated lesions at higher arsenic concentrations (Table 3). In the cancer hospital series, 44% of poorly differentiated lesions were from a thana where the estimated arsenic concentration was 100 μg/L or greater.

This was examined further, restricting the population to those with squamous histology. In this analysis, the classification of arsenic concentration was extended to allow more detailed examination at higher levels. A strong relation was found between undifferentiated lesions and increasing arsenic concentration in well water in the thana of residence in those from the cancer hospital and overall, but not in the smaller number of cases from the community medical service (Table 4). The mean arsenic concentration in those with well differentiated histology was significantly lower (53.9 μg/L SD = 79.0) than in those with poorly differentiated (100.7 μg/L SD = 107.6) F = 60.0 *p* < 0.001. In a multilevel logistic regression, taking account of clustering within thana, and allowing for the source of the specimen (CH or CMS), age, and year of diagnosis, a strong dose response was found, with the odds of poorly differentiated squamous cell carcinoma increasing with higher arsenic concentrations (Table 5).

## 5. Discussion

This analysis did not show any overall excess of cervical malignancy at higher concentrations of arsenic. It did, however, find that among those with a histological diagnosis of squamous cell carcinoma, those from high arsenic areas were more likely to have lesions classified as poorly differentiated. In considering this result, it is important to consider the possibilities of both false positive and false negative results.

There are essentially no data against which to compare the results of the current study. The only paper identified that addressed the relation between arsenic in drinking water and cervical cancer (in women in Patna, India) reported that mean blood arsenic concentration was higher, at 100.7 ppb, in 51 cervical cancer patients than in 48 healthy referents (arsenic concentration not given) [6]. The IARC reviews in 2004 [1] and 2008 [2] included only one study that had cervical cancer as an endpoint, with six deaths [5].

The current study has a number of important weaknesses. There is no record of whether or not a woman drank well water nor, if she did, whether the well from which she drew the water had arsenic concentration close to the levels measured by the British Geological Survey (BGS) [7] for her thana of residence. Within the BGS data, there was a great deal of variation in arsenic concentration between wells in the same thana, and it is quite possible that the woman’s source of drinking water had arsenic concentrations very different from the thana mean used in this analysis. Such misclassification of exposure would tend to bias the estimate of risk towards the null, and so, perhaps, masking a true increase in risk (and resulting in a false negative finding). However, the use of estimates based on thana were sufficiently sensitive to demonstrate a strong dose response for renal cancers [17]. The second important limitation was the comparison series available here. In previous studies using this approach, an unmatched case-referent analysis had been used, with benign lesions defining the comparison (referent) group. In the present study, there were problems in both series with using the benign diagnoses as a referent. The cancer hospital had recently expanded and was able to extend its service to those without proven cancer: it seemed likely that such patients would be drawn disproportionately from those living close to Dhaka, and this was confirmed by inspection of the residence of women from whom the benign specimens were taken in the CH series. This differed markedly from the wide distribution of malignant cases across the whole of Bangladesh and constituted an inappropriate comparison population for an exposure highly determined by geography. The very high proportion of benign lesions found in the CMS series also raised questions about the representativeness of the internal referent group for this series. Given that there is no government screening program, and that less than 1% of the population is reported to have a pap smear [12], it is at least possible that women with symptoms taking advantage of screening clinics may have been more highly educated (and so aware of screening) or have come from more affluent homes, where the cost of additional tests was not a deterrent. It was not practicable to reclassify all 30,050 in the CMS series into “screening” and “clinical indication”, although this was achieved for those with squamous cell histopathology. In 41% of these cases (310/748), the referring gynaecologist had specified concerns about cancer on the requisition. It may be assumed that the proportion was very much lower in those found to be benign.

While an unbiased internal comparison would have been preferable, because of these concerns about the appropriateness of the internal referent groups, an additional external referent group was used to examine further whether there was a higher risk of cervical cancer, of any type, at higher arsenic concentrations in drinking water. Using this external referent series, which could be applied equally to both case series, it was evident that, unlike renal cancers [17], cervical cancers were not drawn disproportionately from thanas with high arsenic concentrations.

An additional limitation was that we had very little information on other potentially causal factors in cervical cancer and so cannot fully test the possibility of confounding. We do not know how many of these women had HPV infection (although presumably many did) and do not know their parity, oral contraceptive use, or smoking habit. There seems little reason to suppose HPV infection would differ with arsenic concentration or that factors associated with the natural history of cervical cancer post HPV infection, such as smoking and oral contraceptive use, would differ systematically with arsenic concentration and so importantly mask a true effect. Nevertheless, given these limitations, it is possible that the data available are too insensitive to detect a relation between cervical cancer and arsenic exposure and that our conclusion of no overall effect is wrong. However, we have no evidence on which to reject the null hypothesis.

We next need to consider whether the strong positive relation between arsenic concentration and a poorly differentiated lesion in those with squamous cell carcinoma might result from bias or confounding. For this analysis, the referent group used was an internal one: those with well differentiated lesions. If the higher grade is a reflection of longer standing disease, it might be, for example, that those from high arsenic areas were for some reason more likely to delay presentation, although the absence of a difference in age would not support that explanation. Rather than reflecting delay, it may be that the arsenic related cancer was poorly differentiated from the outset: we do not know the stage of disease at diagnosis. The strength of the result may be weakened by being present only in the CH series, but the number with poorly differentiated lesions in the CMS series was small (24), and there was perhaps too little power in that series to overcome chance variation. Importantly, this difference is unlikely to be due to reader error: the reading and interpretation of all specimens was carried out blind to arsenic exposure under the guidance of the same pathologist for both series. Although the name of the thana in which the patient lived would not have been hidden, the pathologist (MGM) would at that time have had no knowledge of the arsenic estimated for each thana.

There is some consensus that, in appropriately treated invasive squamous cell cervical cancer, histological grade is of little prognostic significance [19,20]. In a country such as Bangladesh, where diagnosis and treatment of cancer are not widely available, the natural history of poorly differentiated lesions may differ from well differentiated ones to a degree not seen in well treated cases. The results of this study, with no increase in incidence of cervical cancer with estimated arsenic concentration, but with a marked trend to a higher grade (poorly differentiated) at higher arsenic concentrations, cannot offer insight into mechanisms, but only demonstrate that arsenic does affect the disease process. The effect of arsenic may prove to be to promote or act as a co-carcinogen in cancers initiated, as elsewhere, by high risk HPV.

The results reported here, together perhaps with that of Kumar et al. [6], suggest that high concentrations of arsenic are biologically active in the development of squamous cell cancer of the cervix, and as such would warrant systematic investigation. Given the limited data on both HPV infection and on the proportion of cervical cancer cases with such infection in Bangladesh, a large-scale trial to assess the efficacy of screening in areas with both high and low arsenic concentration in well water, with urinary arsenic measurement and HPV characterization in cases and referents, would contribute greatly to knowledge of cervical cancer causality and management in Bangladesh and of the role of arsenic in its evolution.

## 6. Conclusion

A high proportion of Bangladeshis have spent many years drinking water containing >50 μg/L of arsenic. Evidence that arsenic from well water is implicated in cancer grade suggests a need for further investigation of mechanisms and co-factors and the introduction of cervical screening in high arsenic areas.

## Figures and Tables

**Table 1:** Histopathology of malignant cases with mean age and year of diagnosis.

	Cancer Hospital				Community Medical Service			
	N	% within Malignant	Mean Age (SD)	Mean Year (SD)	N	% within Malignant	Mean Age (SD)	Mean Year (SD)
Benign	85	-	38.8 (9.3)	2013.5 (1.2)	28,345	-	43.5 (9.4)	2010.0 (3.1)
Dysplasia (CIN)	50	-	44.9 (10.1)	2012.6 (2.1)	806	-	43.1 (9.6)	2009.9 (1.9)
Squamous cell:								
well differentiated	2913	87.5	48.9 (11.2)	2007.5 (5.1)	724	80.5	47.0 (11.0)	2010.8 (4.1)
poorly differentiated	163	4.9	49.0 (11.9)	2005.3 (4.7)	24	2.7	48.4 (10.4)	2009.8 (5.0)
Adenocarcinoma	206	6.2	44.5 (11.2)	2007.3 (5.2)	108	12.0	48.5 (9.9)	2010.6 (4.2)
Other malignancy	47	1.4	49.0 (12.2)	2010.3 (4.1)	43	4.8	46.5 (10.7)	2011.9 (4.1)
**All malignant**	3329	100.0	48.7 (11.3)	2007.4 (5.1)	899	100.0	47.7 (10.8)	2010.8 (4.1)
**Total**	3464		48.4 (11.4)	2007.6 (5.2)	30,050	100.0	47.7 (10.8)	2010.8 (4.1)
			*p* = 0.000	*p* = 0.000			*p* = 0.000	*p* = 0.000

**Table 2:** Arsenic concentration by diagnosis.

Arsenic Concentration (μg/L)											Compared with Benign Cervix	Compared with Benign Renal
	<10		10 < 50		50 < 100		≥100		Overall		Test for Linearity	Test for Linearity
	N	%	N	%	N	%	N	%	N	%	*p* =	*p* =
**Cancer Hospital** *												
Benign	13	15.3	43	50.6	15	17.6	14	16.5	85	100	-	-
Dysplasia (CIN)	15	30.0	16	32.0	7	14.0	12	24.0	50	100	0.856	0.846
Squamous cell:												
well differentiated	1144	39.3	907	31.1	382	13.1	480	16.5	2913	100	0.017	0.005
poorly differentiated	33	20.2	33	20.2	26	16.0	71	43.6	163	100	0.002	0.000
Adenocarcinoma	79	38.3	49	23.8	29	14.1	49	23.8	206	100	0.408	0.661
Other malignant	13	27.7	19	40.4	9	10.1	6	12.8	47	100	0.292	0.543
All malignant	1269	38.1	1008	30.3	446	13.4	606	18.2	3329	100	0.052	0.031
All	1297	37.4	1067	30.8	468	13.5	632	18.2	3464	100	-	-
**Community Medical Service** **												
Benign	9172	32.4	8970	31.6	3774	13.3	6429	22.7	28,345	100	-	-
Dysplasia (CIN)	250	31.0	259	32.1	112	13.9	185	23.0	806	100	0.544	0.963
Squamous cell:												
well differentiated	215	29.7	238	32.9	116	16.0	155	21.4	724	100	0.509	0.930
poorly differentiated	8	33.3	11	45.8	1	4.2	4	16.7	24	100	0.340	0.341
Adenocarcinoma	42	38.9	35	32.4	13	12.0	18	16.7	108	100	0.071	0.109
Other malignant	14	32.6	22	51.2	6	14.0	1	2.3	43	100	0.020	0.027
All malignant	279	31.0	306	34.0	136	15.1	178	19.8	899	100	0.495	0.575
All	9701	32.3	9535	31.7	4022	13.4	6792	22.6	30,050	100	-	-
**Renal Lesions**												
Females with benign renal lesions	79	35.6	58	26.1	28	12.6	57	25.7	222	100	-	-

* χ^2^ = 120.96 *p* = 0.000, ** χ^2^ =26.62 *p* = 0.032.

**Table 3:** Logistic regression * for well differentiated squamous cell, poorly differentiated squamous cell and all malignancies by arsenic concentration with benign renal lesions as the comparison group.

Arsenic Concentration (μg/L)	Squamous Cell								All Malignancies			
	Well Differentiated				Poorly Differentiated							
	Cancer Hospital		Both Series		Cancer Hospital		Both Series		Cancer Hospital		Both Series	
	OR	95% CI	OR	95% CI	OR	95% CI	OR	95% CI	OR	95% CI	OR	95% CI
<10	1	-	1	-	1	-	1	-	1	-	1	-
10 < 50	1.07	0.61–1.86	1.15	0.67–1.96	1.37	0.51–3.69	1.52	0.60–3.86	1.05	0.61–1.81	1.18	0.70–2.01
50 < 100	0.90	0.45–1.81	1.04	0.51–2.10	3.42	1.09–10.70	2.74	0.91–8.23	0.99	0.49–1.98	1.10	0.55–2.20
≥100	0.42	0.22–0.79	0.46	0.24–0.87	4.98	1.92–12.96	3.74	1.51–9.23	0.49	0.26–0.91	0.51	0.27–0.97

* Allowing for clustering within thana.

**Table 4:** Proportion of squamous cell lesions that are poorly differentiated by arsenic concentration.

Arsenic Concentration	Cancer Hospital			Community Medical Service			All		
(μg/L)	n	%	N	n	%	N	n	%	N
<10	33	2.8	1177	8	3.6	223	41	2.9	1400
10 < 50	33	3.5	940	11	4.4	249	44	3.7	1189
50 < 100	26	6.4	408	1	0.9	117	27	5.1	525
100 < 200	37	11.3	328	4	3.7	107	41	9.4	435
≥200	34	15.2	223	0	0.0	52	34	12.4	375
Overall	163	5.3	3076	24	3.2	748	187	4.9	3834
Test for linearity	χ^2^ = 80.75 *p* = 0.000			χ^2^ = 1.57 *p* = 0.210			χ^2^ = 60.82 *p* = 0.000		

**Table 5:** Logistic regression for histological diagnosis of poorly differentiated squamous cell carcinoma, allowing for clustering within thana (N = 3824).

Arsenic Concentration (μg/L)	Odds Ratio	95% Confidence Interval
<10	1	-
10 < 50	1.58	0.89–2.80
50 < 100	2.61	1.35–5.00
100 < 200	4.56	2.47–8.42
≥200	8.11	4.08–16.15
Source of specimen:		
cancer hospital	1.97	1.20–3.21
Age (continuous)	1.01	0.99–1.02
Year of diagnosis:		
Before 2010	3.26	2.28–4.66
